# Supplementary material for: The gut-brain vagal axis governs mesolimbic dopamine dynamics and reward events
Source: Sci Adv. 2026 Jan 30;12(5):eadz0828. doi: 10.1126/sciadv.adz0828 (PMC12857734; doi:10.1126/sciadv.adz0828)
Supplement: Supplementary file 1 — Figs. S1 to S7 Tables S1 to S3 [file sciadv.adz0828_sm.pdf]

Science Advances
AAAS

Supplementary Materials for

The gut-brain vagal axis governs mesolimbic dopamine dynamics and reward events

Oriane Onimus *et al.*

Corresponding author: Giuseppe Gangarossa, giuseppe.gangarossa@u-paris.fr

Sci. Adv. **12**, eadz0828 (2026)
DOI: 10.1126/sciadv.adz0828

This PDF file includes:

Figs. S1 to S7
Tables S1 to S3

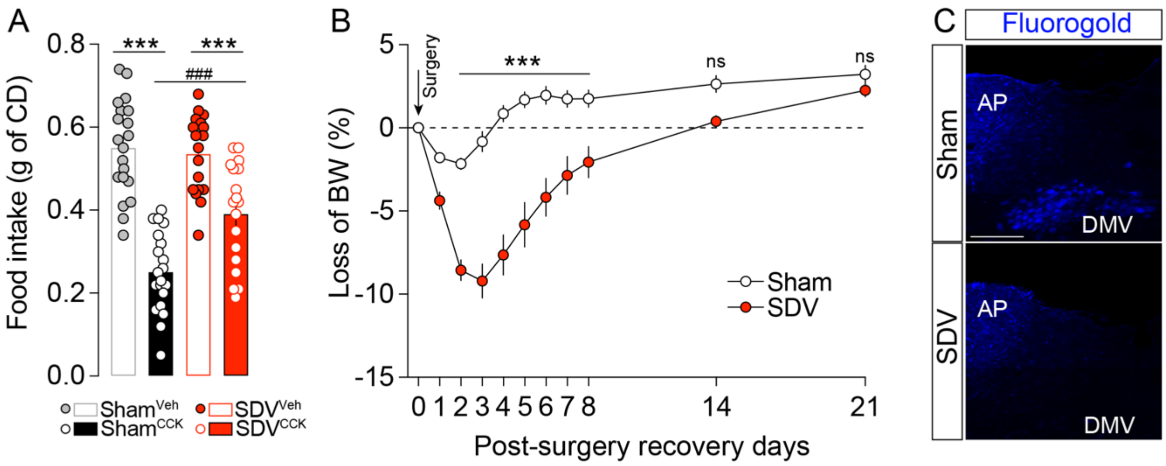

Figure S1: Validation of the SDV model. (**A**) 1h of food intake (chow diet, CD) in fasted Sham (n=20) and SDV (n=18) mice following administration (i.p.) of vehicle (Veh) or CCK-8S (10 µg/kg). (**B**) Measurement of body weight loss (%) in Sham (n=20) and SDV (n=20) mice during the post-surgery recovery period (3 weeks). (**C**) Fluorogold staining in the brainstem (AP, area postrema; DMV, dorsal motor nucleus of the vagus) of Sham and SDV mice. Scale bar: 100 µm. Statistics: ***$p<0.001$, $^{###}p<0.001$ for specific comparisons. Two-way ANOVA (A, B).

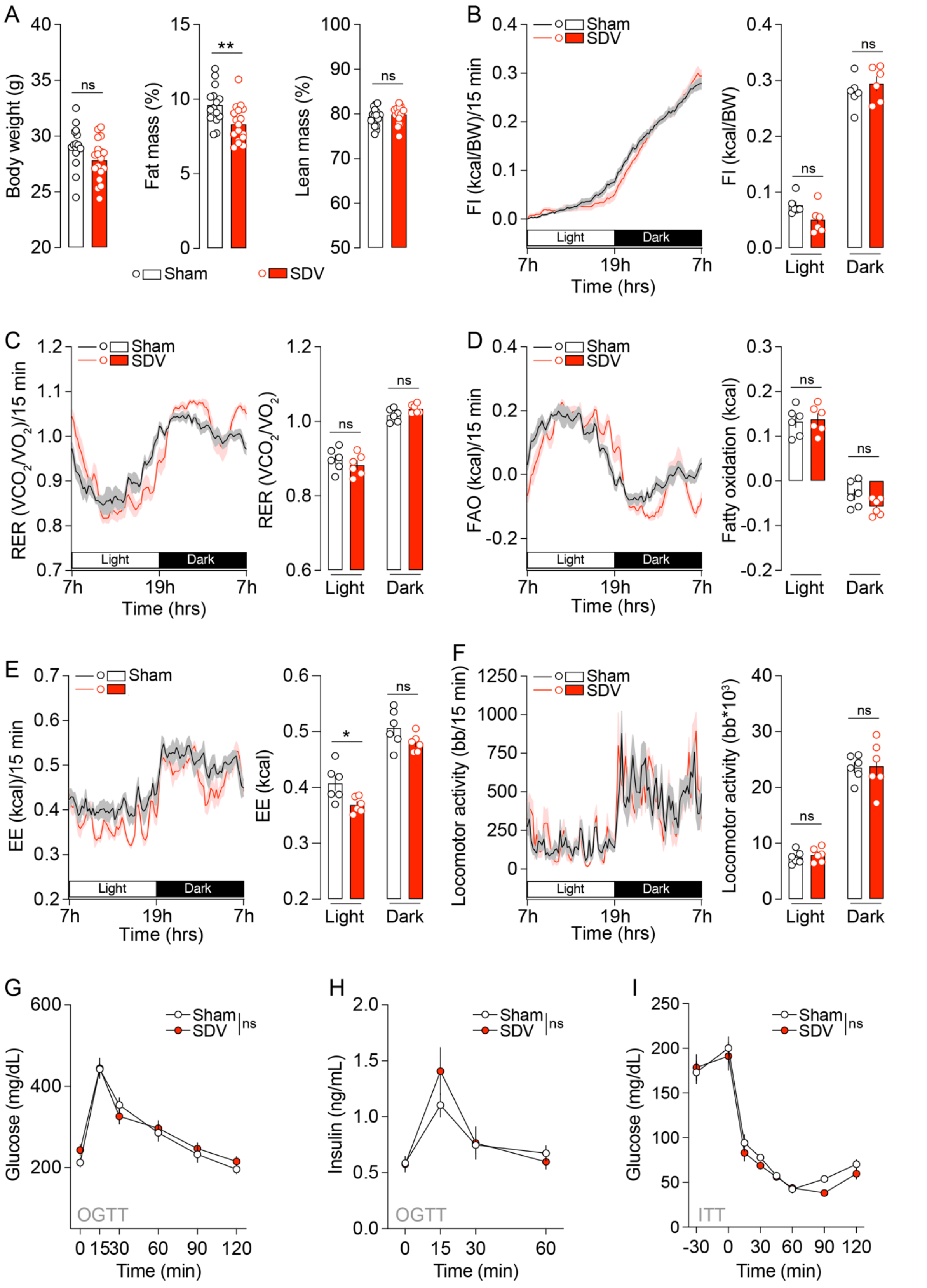

Figure S2: Metabolic profile of Sham and SDV mice. (**A**) Body weight, fat and lean mass of Sham (n=15) and SDV (n=18) mice. (**B-F**) Measurements of food intake (FI), respiratory exchange ratio (RER), fatty acid oxidation (FAO), energy expenditure (EE) and locomotor

activity in Sham (n=6) and SDV (n=6) mice. (**G-H**) Glucose and insulin profiles following an oral glucose tolerance test (OGTT) in Sham (n=10) and SDV (n=9) mice. (**I**) Glucose profile following an insulin tolerance test (ITT) in Sham (n=9) and SDV (n=8) mice. Statistics: *$p<0.05$ for specific comparisons. Student's t-test (A); Two-way ANOVA (B, C, D, E, F, G, H, I).

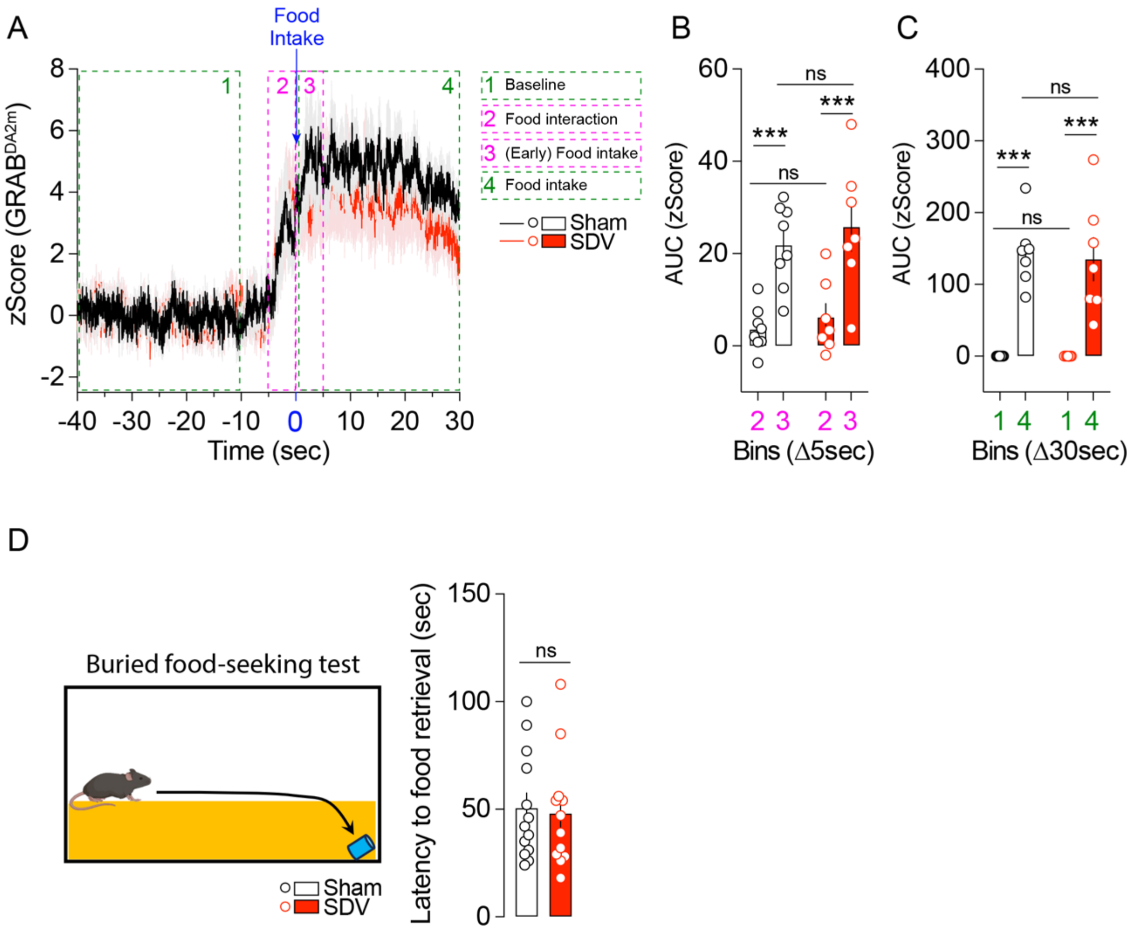

Figure S3: SDV mice show intact DA dynamics during palatable food interaction and consumption (acute exposure) as well as olfactory functions. (**A, B, C**) *In vivo* DA dynamics ($GRAB^{DA2m}$) during palatable food (HFD pellet) interaction (purple dashed rectangle 2) and consumption [purple dashed rectangle 3 for early food intake (5 sec), and green dashed rectangle 4 for food intake (30 sec)] in the NAc of Sham (n=8) and SDV (n=7) mice using the $GRAB^{DA2m}$ biosensor. (**D**) Latency of food retrieval (HFD pellet) of Sham (n=13) and SDV (n=12) mice in the buried food-seeking test. Statistics: ***p<0.001 for specific comparisons. Two-way ANOVA (B, C); Student's t-test (D).

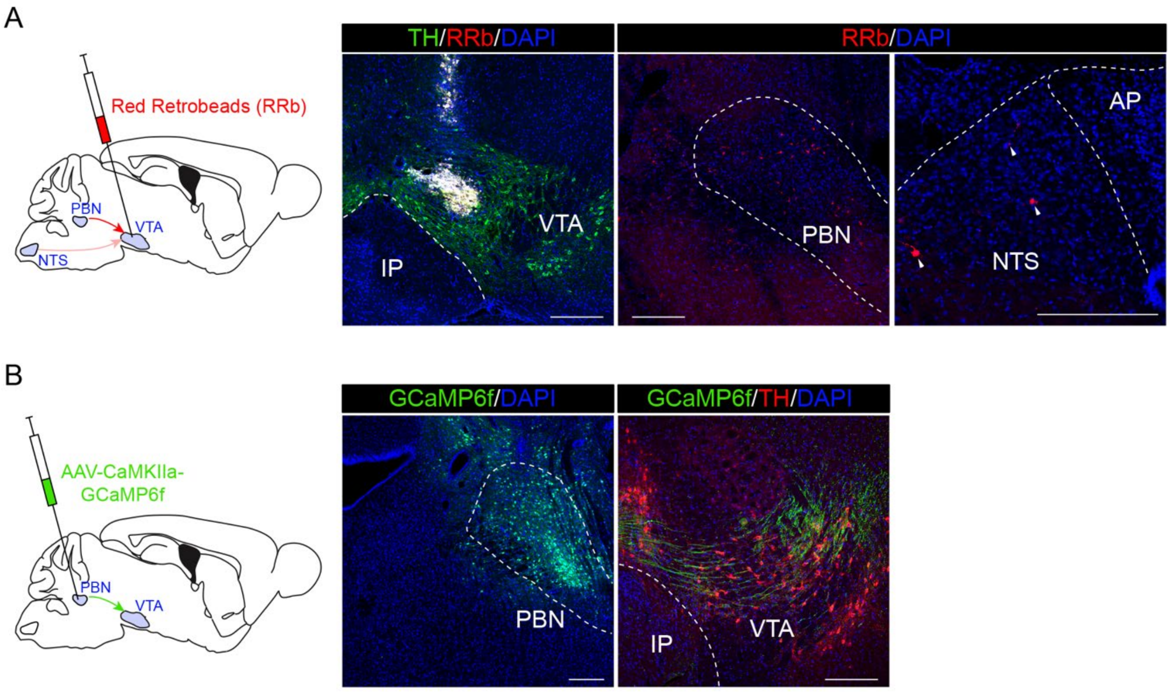

Figure S4: Connections from brainstem nuclei to the VTA. (**A**) Drawing illustrates the retrograde tracing approach [Red Retrobeads (RRb)]. Images show the injection site (VTA) and RRb-positive neurons in the PBN and NTS. (**B**) Drawing illustrates the anterograde viral (AAV-CaMKIIa-GCaMP6f) strategy. Images show the injection site (PBN) and GCaMP6f-positive terminals in the VTA. Scale bars: 250 μm.

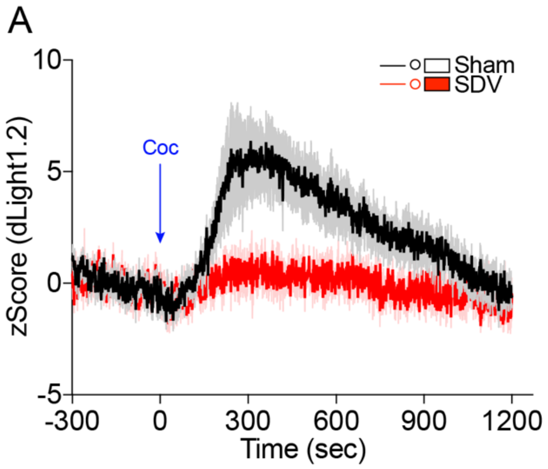

Figure S5: *In vivo* DA transients following administration of cocaine. (**A, B**) *In vivo* DA dynamics during cocaine-induced DA release/accumulation in the NAc of Sham (n=6) and SDV (n=5) mice using the dLight1.2 biosensor. Statistics: *$p<0.05$ and ***$p<0.001$ for specific comparisons. Two-way ANOVA (B).

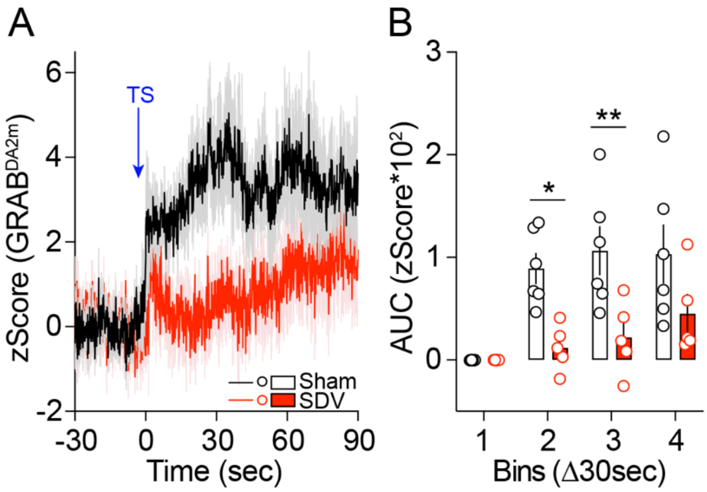

Figure S6: *In vivo* DA transients following a tail suspension test. (**A, B**) *In vivo* DA dynamics (GRAB^{DA2m}) during a tail suspension (TS)-induced DA release/accumulation in the NAc of Sham (n=6) and SDV (n=5) mice. Statistics: *$p<0.05$ and **$p<0.01$ for specific comparisons. Two-way ANOVA (B).

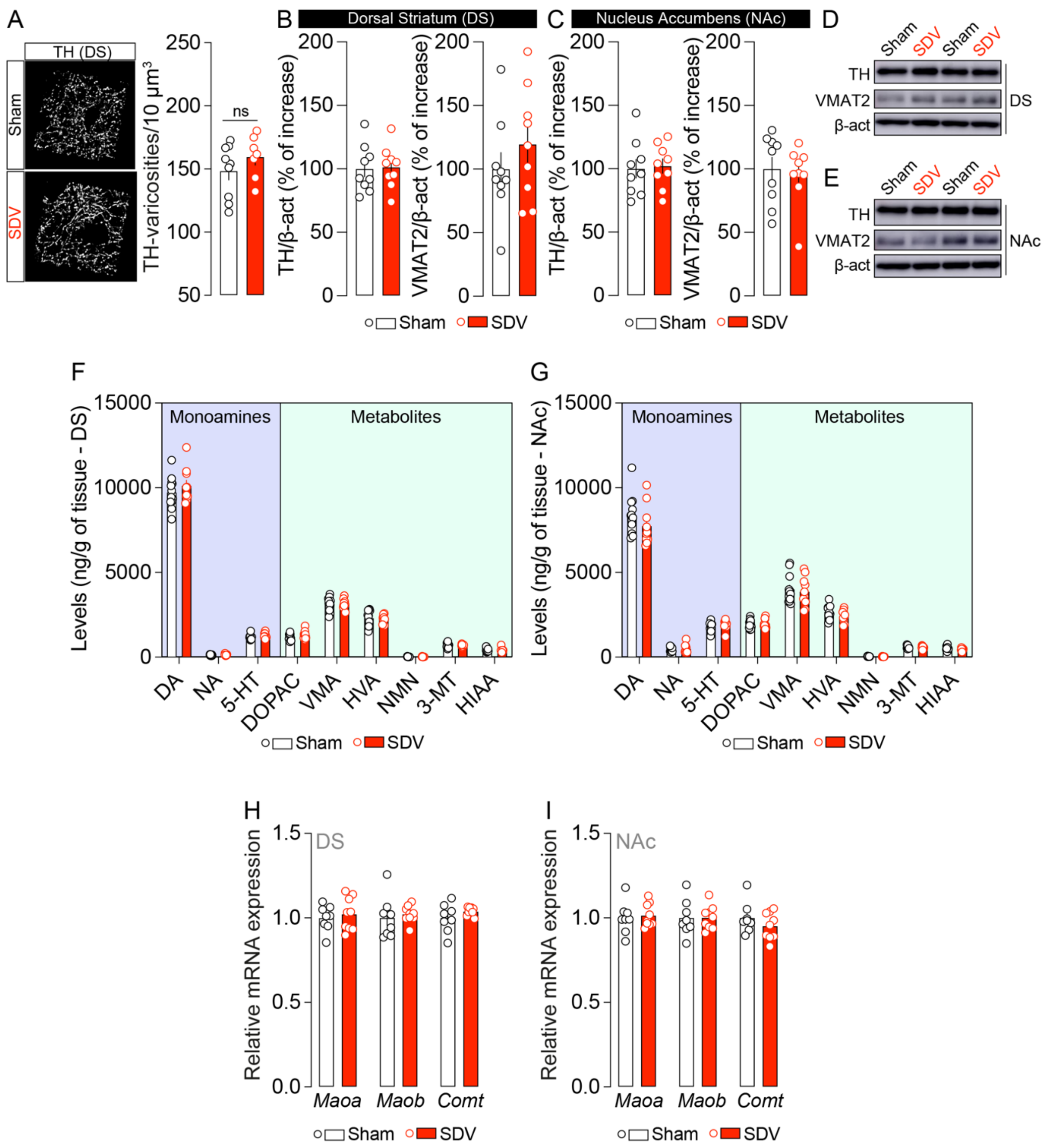

Figure S7: The integrity of the gut-brain vagal axis is not necessary for DA synthesis and metabolism. (**A**) 3D reconstruction and computational segmentation of VTA-projecting TH-positive varicosities in the NAc of Sham (n=9) and SDV (n=7) mice. (**B-E**) Expression and representative blots of TH and VMAT2 in the DS (**B, D**) and NAc (**C, E**) of Sham (n=9) and SDV (n=9) mice. (**F, G**) Quantifications of monoamines [dopamine (DA), noradrenaline (NA), serotonin (5-HT)] and their metabolites (DOPAC, VMA, HVA, NMN, 3-MT and HIAA) in the DS and NAc of Sham (n=12) and SDV (n=10) mice. (**H, I**) Relative expression of *Maoa*, *Maob*, *Comt* in the DS and NAc of Sham (n=8) and SDV (n=9) mice. Statistics: Student's t-test (A, B, C, F, G, H, I).

Primers of Figs. 4, 5, 8 and Fig. S7		
**Gene**	**Sens**	**5' → 3'**
Arc	Forward	AAGTGCCGAGCTGAGATGC
	Reverse	CGACCTGTGCAACCCTTTC
Comt	Forward	CTGGGGGTTGGTGGCTATTG
	Reverse	CCCACTCCTTCTCTGAGCAG
Drd1	Forward	ATCGTCACTTACACCAGTATCTACAGGA
	Reverse	GTGGTCTGGCAGTTCTTGGC
Drd2	Forward	TGAACAGGCGGAGAATGG
	Reverse	CTGGTGCTTGACAGCATCTC
Egr1	Forward	CGAACAACCCTATGAGCACCTG
	Reverse	CAGAGGAAGACGATGAAGCAGC
Egr3	Forward	CCGGTGACCATGAGCAGTTT
	Reverse	TAATGGGCTACCGAGTCGCT
Fos	Forward	CGGGTTTCAACGCCGACTA
	Reverse	TTGGCACTAGAGACGGACAGA
Fosb	Forward	TTTTCCCGGAGACTACGACTC
	Reverse	GTGATTGCGGTGACCGTTG
Gria1	Forward	GTCCGCCCTGAGAAATCCAG
	Reverse	CTCGCCCTTGTCGTACCAC
Grin1	Forward	AGAGCCCGACCCTAAAAAGAA
	Reverse	CCCTCCTCCCTCTCAATAGC
Grm1	Forward	CATACGGAAAGGGGAAGTGA
	Reverse	AAAAGGCGATGGCTATGATG
Maoa	Forward	GTATGGAAGGGTGATTCGGCA
	Reverse	ACTGCACCTTCCATGTAGCC
Maob	Forward	ATGAGCAACAAAAGCGATGTGA
	Reverse	TCCTAATTGTGTAAGTCCTGCCT
Pdyn	Forward	CTCCTCGTGATGCCCTCTAAT
	Reverse	AGGGAGCAAATCAGGGGGT
Penk	Forward	CATGAAACGGTACGGAGGCT
	Reverse	GCCAAGGTGTCTCCCTCATC
Rpl19	Forward	GGGCAGGCATATGGGCATA
	Reverse	GGCGGTCAATCTTCTTGGATT
Scl17a6	Forward	TGGAAAATCCCTCGGACAGAT
	Reverse	CATAGCGGAGCCTTCTTCTCA

Table S1: List of primers used in the study.

Table S2	Nucleus accumbens (NAc)					
	D1-SPN			D2-SPN		
	**Sham** (n=14)	**SDV** (n=14)	**Stats** (*p* value)	**Sham** (n=14)	**SDV** (n=14)	**Stats** (*p* value)
**Passive membrane properties**						
RMP (mV)	-75.86 ± 1.565	-78.64 ± 0.992	0.1155	-79.50 ± 0.789	-77.57 ± 1.357	0.0868
Ri (MOhms)	111.0 ± 16.70	118.1 ± 50.16	0.5714	84.54 ± 9.724	102.6 ± 8.763	* 0.0274
Membrane time constant (ms)	7.953 ± 1.495	7.416 ± 1.436	0.8036	6.047 ± 0.6363	6.657 ± 0.6985	0.4824
Inward rectification ratio	0.7964 ± 0.0280	0.7810 ± 0.0338	0.9820	0.8461 ± 0.0251	0.8009 ± 0.0224	0.2100
**Action potential properties**						
Rheobase	174.7 ± 22.85	168.4 ± 23.15	0.8132	254.9 ± 26.48	182.4 ± 17.59	* 0.0156
AP threshold (pA)	-34.49 ± 2.232	-35.60 ± 1.331	>0.9999	-35.00 ± 1.207	-36.21 ± 0.7837	0.5714
Delay to 1st spike (ms)	510.9 ± 30.25	449.9 ± 29.08	0.1167	571.4 ± 27.11	507.4 ± 39.33	0.2852
AP Amplitude (pA)	80.32 ± 2.161	84.68 ± 2.620	0.0690	84.67 ± 1.453	82.99 ± 1.863	0.3519
AP rise time (ms)	0.4111 ± 0.0143	0.4332 ± 0.0253	0.4752	0.4086 ± 0.0204	0.4071 ± 0.0129	0.9729
AP rise/decay ratio	1.299 ± 0.0667	1.438 ± 0.0901	0.2700	1.159 ± 0.0384	1.351 ± 0.0677	* 0.0359
**Action potential trains**						
I/O gain function	0.2618 ± 0.0161	0.2811 ± 0.0096	0.0776	0.2264 ± 0.0170	0.2694 ± 0.0088	* 0.0404
Firing rate at +50 pA	15.33 ± 0.986	17.24 ± 0.567	* 0.0226	13.33 ± 0.937	16.00 ± 0.484	* 0.0310
SFA ratio	1.272 ± 0.1957	1.156 ± 0.0944	0.9820	0.981 ± 0.0364	1.019 ± 0.0726	0.6260

Table S2: Cell type-specific electrophysiological parameters measured in the NAc of Sham and SDV mice.

Table S3	Dorsal striatum (DS)					
	D1-SPN			D2-SPN		
	Sham (n=13)	SDV (n=12)	Stats (*p* value)	Sham (n=14)	SDV (n=13)	Stats (*p* value)
**Passive membrane properties**						
RMP (mV)	-80.08 ± 0.780	-80.00 ± 0.674	0.8611	-80.93 ± 1.495	-79.54 ± 1.153	0.1338
Ri (MOhms)	62.55 ± 4.058	75.47 ± 7.525	0.1683	70.55 ± 3.298	66.96 ± 6.778	0.0850
Membrane time constant (ms)	5.086 ± 0.415	5.093 ± 0.518	0.8938	6.066 ± 0.530	6.703 ± 1.202	0.8300
Inward rectification ratio	0.8705 ± 0.0304	0.8816 ± 0.0276	0.9787	0.8280 ± 0.0225	0.8519 ± 0.0278	0.4583
**Action potential properties**						
Rheobase	298.5 ± 22.14	276.7 ± 27.10	0.4785	311.6 ± 28.28	299.0 ± 22.79	0.6500
AP threshold (pA)	-39.33 ± 1.250	-38.72 ± 1.009	0.9362	-37.63 ± 0.9604	-40.27 ± 1.263	0.1021
Delay to 1st spike (ms)	501.7 ± 37.56	477.0 ± 39.84	0.5033	501.0 ± 32.58	495.9 ± 32.55	0.9810
AP Amplitude (pA)	93.96 ± 1.820	93.98 ± 2.783	0.9048	90.85 ± 1.761	96.45 ± 1.602	* 0.0427
AP rise time (ms)	0.4023 ± 0.0110	0.3817 ± 0.0143	0.8202	0.3671 ± 0.0175	0.3890 ± 0.00850	0.6066
AP rise/decay ratio	2.516 ± 0.0969	2.970 ± 0.2613	0.1519	2.899 ± 0.1653	3.123 ± 0.1264	0.1159
**Action potential trains**						
I/O gain function	0.2450 ± 0.0187	0.2746 ± 0.0179	0.2993	0.2171 ± 0.01407	0.2400 ± 0.01121	0.2836
Firing rate at +50 pA	14.67 ± 1.0350	16.11 ± 0.9773	0.2832	13.24 ± 0.7053	14.36 ± 0.5042	0.2468
SFA ratio	0.8981 ± 0.0548	0.8707 ± 0.0263	0.9056	0.8868 ± 0.0342	0.9351 ± 0.0331	0.2800

Table S3: Cell type-specific electrophysiological parameters measured in the DS of Sham and SDV mice.
